# 

**Published:** 1870-05

**Authors:** 


### CONTENTS:

### ORIGINAL COMMUNICATIONS.

ARTICLE I.—The Discovery of Cells with Fibres, Dentine at the Junction of the Enamel and Cementum. By Geo. B. Harriman, D. D. S., ........ 1

ARTICLE II.—Mechanical Dentistry. By J. Z. Hoffer, D.D.S. ........ 5

ARTICLE III.—Recent Views on Digestion. By J R. Bromwell, D.D.S ........ 14

### SELECTED ARTICLES.

ARTICLE IV.—On the use of Chloroform in Infantile Convulsions. By David Mackintosh, M.D., L.R.C.S ........ 22

ARTICLE V.—Irregularities of the Teeth. By H. D. Ross, D.D.S. ........ 34

### MONTHLY SUMMARY.

Hydrate of Chloral, ........ 39

Death from Chloroform. Fatal Tooth Pulling, ........ 39

Almost Complete Ossification of the Human Body, ........ 40

New Operations on the Lower Jaw. Lithotrity, ........ 41

How to Cure a Cold, ........ 41

A Snake in the Stomach, ........ 42

Death Following the Application of Creasote to a Carious Tooth, ........ 43

Squills as a Poison for Rats, ........ 43

Influence of Sewing Machines on Health, ........ 44

Bichloride of Methylene, ........ 44

Doses for Hypodermic Use ........ 45

### EDITORIAL DEPARTMENT.

The Southern Dental Association Meeting at New Orleans, ........ 46

Monument to Dr. Horace Wells, the Discoverer of Aneasthesia, ........ 47

